# Bog bacterial community: data from north-western Russia

**DOI:** 10.3897/BDJ.12.e118448

**Published:** 2024-08-21

**Authors:** Ivan Zubov, Dmitrij Shpanov, Tamara Ponomareva, Andrey Aksenov

**Affiliations:** 1 N. Laverov Federal Center for Integrated Arctic Research of the Ural Branch of the Russian Academy of Sciences, Arkhangelsk, Russia N. Laverov Federal Center for Integrated Arctic Research of the Ural Branch of the Russian Academy of Sciences Arkhangelsk Russia; 2 Northern (Arctic) Federal University named after M.V. Lomonosov, Arkhangelsk, Russia Northern (Arctic) Federal University named after M.V. Lomonosov Arkhangelsk Russia

**Keywords:** barcode amplicon sequencing, 16S rRNA gene, prokaryote, high-moor peat, bog, biodiversity, north-western Russia

## Abstract

Wetlands occupy up to 35% of the boreal biome in Russia, according to various estimates. Boreal bogs are global carbon sinks, accounting for more than 65% of the soil carbon stored in the wetland ecosystems of the world. The decomposition of plant residues is one of the most important components of the carbon cycle in wetland systems, while the violation of their fragile balance due to climate change increases the rate of mineralisation of organic matter and releases large amounts of carbon to the atmosphere. The biochemical processes occurring in a peat deposit determine the intensity of the destruction of organic matter and gas exchange. However, the microbial communities of the boreal ombrotrophic bogs, regulating those processes, are poorly studied.

Hence, a study of the prokaryote communities of the peat deposits of the southern White Sea coastal ombrotrophic bogs (mostly spread in north-western Russia) was carried out. The taxonomic composition of archaea and bacteria sampled from the deposit’s depth of 0–310 cm was studied using high-throughput sequencing of V4 sites of 16S rRNA gene by Illumina technology. As a result, 105 species belonging to 19 phylums were identified. The dominant specific phyla were *Pseudomonadota*, *Acidobacteriota* and *Verrucomicrobiota*, the non-specific phylum being *Desulfobacterota*. Various groups of methanogenic, methylotrophic and nitrogen-fixing microorganisms were identified. Shannon's biodiversity ranged from 3.5 to 4.6 and ChaO1 - from 232 to 351, decreasing within the depth.

## Introduction

Wetlands are biologically productive ecosystems, having a significant impact on carbon cycling, water storage and greenhouse gas emissions. The land area occupied by them exceeds 4 million km^2^, with 80% of the area located in the temperate zone of the Northern hemisphere (Wieder and Vitt 2006).

The wetlands of the boreal biome are estimated to reach 35 % of the area, with bogs being the most common among them (Vompersky et al. 2012, Parfenova et al. 2016). Such a large area is characterized by high autonomy, and specificity of the environment. Moreover, these territories are mostly preserved in a natural undisturbed state (*Ipatov and Kirikova 1997, Sokolov 2000, Wieder and Vitt 2006*). The study of the properties of bog complexes can deliver the fresh data on bacterial communities, further extrapolating it to a wider area.

Biochemical processes occurring in a peat deposit determine the intensity of the degradation of organic matter and of gas exchange. These processes depend on functioning of microbial communities, especially bacteria - the most abundant group of micro-organisms in a peat deposit (Volkova et al. 2022). At the same time, bogs are a highly specific environment characterized by low pH (< 4.0) and low nutrients content, which contributes to the formation of unique microbiomes (Quatrini and Johnson 2018). This is the reason for the numerous studies on bog complexes to happen worldwide (Serkebaeva et al. 2013, Dobrovol’skaya et al. 2019, Apkin 2021). Most authors, especially those investigating objects in the Arctic zone, focus on describing only acrotelm and neglect to characterize them throughout the deposit depth.

The advent and development of next-generation sequencing technology (NGS) and, in particular, the spread of Illumina-SOLEXA method allowed the mass production of short reads of 16S rRNA gene clone amplified libraries, which greatly improved the understanding of the composition of microbial communities in natural biomes (Nikitin et al. 2022).

The study investigates taxonomic and quantitative characteristic of prokaryotes throughout the deposit down to 310 cm depth. The studied peat deposit (ombrotrophic bog complex) is located in the European part of the boreal zone of the Russian Federation.

## Material and methods

### Object of research

Being part of the bog system «Ilasskoe», the Ilas Bog Massif has been selected as the study plot, representing the southern White Sea coastal ombrotrophic bogs. It has a combination of microtopes standing for integrated ridge-hollow-pool and integrated ridge-pool mesotopes with oligotrophic vegetation type on ridges and in hollows. The bog system spans more than 90 km^2^: 17 km from West to East and for almost 7 km from North to South. The plot is located 30 km south-south-west of Arkhangelsk, in the highly boggy part of the northern sub-zone of the taiga, on the watershed of three rivers: Brusovica, Shuhta and Ilas (northern Dvina River Basin).

**Figure 1.**
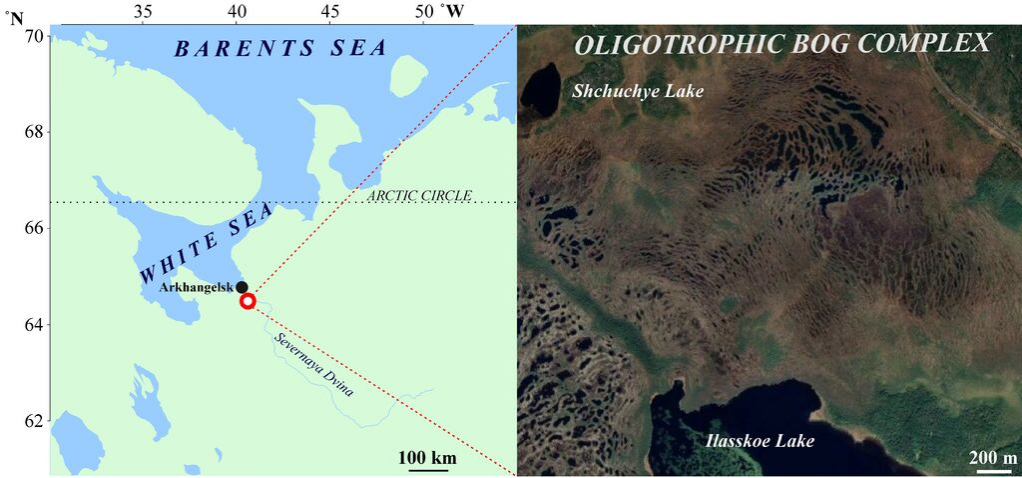
Location of the experimental sites within the Ilas Bog complex.

The climate of the region is temperate cold, slightly continental Atlantic-Arctic with a pronounced influence of the White and Barents Seas. The influence of air mass transfer from the Atlantic Ocean is also noted. The average annual temperature is +1.9˚C, with a maximum in July (16.5˚C) and a minimum in January (-11.6˚C). The mean annual precipitation is 634 mm and 53% of annual precipitation (296 mm) occurs in the warm season (May–September). The average number of days with stable snow cover is 180; the maximum snow depth is observed in March (102 cm). The Ilas Bog Massif area is 595 hectares and is an ombrotrophic bog (Fig. 1).

**Figure 2.**
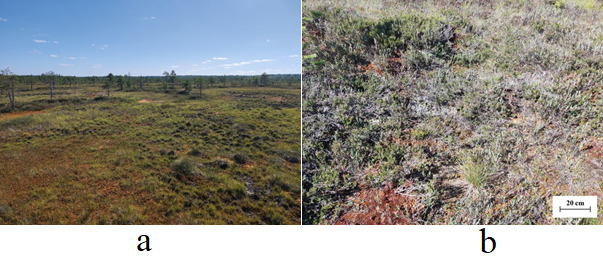
Examples of the ridge-hollow mesotope of Ilas Bog Massif: **a** overview; **b** plant community on the study site.

The study plot is located in the centre of the ridge, which is about 30 cm high, 7-12 m wide and formed perpendicular to the waterflow direction. The ridges’ occurrence is about 20%. This is formed by tree base mounds and peat hillocks. The plant community of the selected site of the ridge belongs to the Scots pine-dwarf shrub-Sphagnum type. The tree layer is represented by rare single Scot pine trees (*Pinussylvestris* f. Litwinovii L.). The crown density of the tree layer on the ridges is 0.2-0.3. Average stand height is 2.4 m, average diameter is 4.3 cm and the average age is 189 years. The shrubs are represented by rare plants of dwarf birch (*Betulanana* L.). The projected cover of the grass-dwarf shrub layer reaches 45%. The grass-dwarf shrub layer is dominated by *Andromedapolifolia* L., *Callunavulgaris* (L.) Hull and *Empetrumhermaphroditum* L. The moss-lichen layer has a projected coverage of up to 80% and is predominantly formatted by *Sphagnumfuscum* H.Klinggr. and lichens of the genus *Cladonia*. An overview of the Ilas Bog Massif ridge-hollow mesotope is given in Fig. 2.

The field observation proves the homogeneous botanical composition of the peat deposit. Its thickness reaches 3.5 m. The underlying rocks are moraine loam and clay. The groundwater level changed during the study period from 0 cm during the period of active snowmelt and heavy precipitation to -33 cm during the summer low-water period (mid-July).

### Peat sampling and probe fixation

The peat was sampled by a layer-by-layer drilling method using a peat sampler made of stainless steel P 04.09 (0.5 m chamber length, 5.0 cm inside diameter by EIJKELKAMP, Netherlands) from a depth of 0-310 cm and was aseptically packed. Analyses were carried out using the integral 10 cm thick samples (mixed from 3 peat cores) (Zubov et al. 2022). The samples for genetic analysis were placed in 1.5 ml microtubes and fixed in cryogenic nitrogen immediately after sampling and were stored at -80°C until the DNA was extracted (Zvyagintsev et al. 1991).

### Total bacterial count (TBC)

The samples were prepared and fixed simultaneously. A soil suspension was prepared at a ratio of 1:100 (1 g of peat per 100 ml of 0.85% NaCl solution). Later, it was treated on the orbital shaker LS 110 («LOIP», Russia) for 30 minutes at 170 RPM for the dissection of large pieces of substrate. The sample was additionally dispersed by the ultrasonic UZV-5.7 («Sapphire», Russia) (3 min, frequency 35 kHz) (Zvyagintsev et al. 1991). After treatment, the suspension was transferred to 15 ml glass vials and fixed by 50% gluteraldehyde to a final concentration of 0.3%. Colouring and counting were carried out no less than 5 days after fixing the sample.

The total number of microorganisms was determined by a luminescent microscopic method with concentration of cells on MCE black mesh membrane filters with a pore size of 0.22 µm («Hawach Scientific», China) (Astashkina 2015). The filter was placed on a substrate of an ashless paper filter with a pore diameter of 1.5-2.5 μm attached to a special holder on a Bunsen flask for a homogeneous cell distribution. After the application of an aliquot suspension of peat in the isotonic solution and fluorescent dye DAPI (up to C = 10 µg/ml) were dyed in the dark (10 min). After incubation, the surplus fluid was removed by a vacuum pump, depositing the cells on the filter. The cells were counted using the fluorescent microscope LUM1 («Altami», Russia), equipped with a high-voltage mercury lamp NBO 100 W, with magnification of 100x (the achromatic lens PL 100x / 1.25 MI). Optical filters used for cell counting were: excited ultraviolet from 395 to 415 nm, emissive blue from 420 to 485 nm. The measurements were carried out by splitting the filters into four pieces, on each part at least 200 cells in 10 fields of vision were considered to obtain data with an error of no more than 20% at 95% confidence level. In order to reduce the time of the action of the excitation radiation on the dyed cells, we photographed the field of vision (Canon EOS 1100D digital camera, connected via optical pho-adapter EF 2,5X-4X). Image processing and cell counting were done through Altami Studio software (3.3.0 (r949c46)).

The humidity of peat and the volume of solutions (the fixator and the isotonic 0.85% NaCl used for dilution) were taken into account to calculate the total number of microorganisms. The resulting value was the number of cells per 1 g of a completely dry substrate (cells/g).

### 16S rRNA Gene metabarcoding

The DNA was extracted from the mixed samples, following the RIAM protocol (Pinaev et al. 2022). Absence of sorption-desorption stage on the silica columns, introduction of high phosphate concentrations in the buffer solutions, preventing DNA sorption on minerals, CTAB DNA extraction, accelerating and simplifying the purification process are the advantages of the method. The gene library was prepared using the standard PCR protocol with straight and reverse primers on the highly variable V4 regions of the 16S rRNA gene (515F-GTGCCAGCMGCCGCGGTAA/ 806R-GGACTACVSGGGTATCTAAT) (Bates et al. 2010) along with linkers and unique indexes.

Deep sequencing of the above-mentioned library of amplicons of the 16S rRNA gene fragment was performed to analyse the taxonomic structure of the soil microbiome. A library using 515F/806R primers, along with linker and unique index, was prepared using the thermocycler T100 (BIO-RAD Laboratories, Heracles, California, USA) containing 0.5 units of high precision DNA polymerase Q5® (New EnglandBioLabs, Ipswich, Massa Chucets, USA), the 1X reactive buffer Q5, 5 μm of each primer, 2 mm dNTP (LifeTechnologies, Carlsbad, California, USA) and 1-5 ng DNA matrices. The PCR programme included a denaturation stage at 94°C for 1 min, 25-cycles amplification of the product (94°C for 30 s, 50°C for 30 s, 72°C for 30 s) and a final elongation at 72°C for 3 min. Further sample preparation and sequencing were carried out in accordance with the Illumina Protocol ("16S Metagenome Sequencing Library Preparation") on the Illumina MiSeq platform (Illumina Inc., San Diego, California, USA).

Initial data processing, including demultiplexing and trimming of the adapter, was performed using Illumina software (Illumina Inc., San Diego, California, USA). The dada2 method (Callahan et al. 2016) was used in R software for noise reduction, read fusion, generation of amplicon sequence variants (ASV) and chimera removal. The ASV taxonomy was classified using the DECIPHER package (Wright 2016). Portions of rDNA v.4 from the SILVA database records (issue 138) (Quast et al. 2012) were extracted to train the classifier. They were further used as a learning set for the LearnTaxa (DECIPHER) function. The ASV classification was performed using the IdTaxa function with a confidence threshold of 70. The phylogenetic tree is based on the SEPP fragment insertion algorithm implemented in the QIIME2 plugin. Biodiversity indices were calculated in the PAST programme (version 3.01). The construction of the histogram was carried out in Excel 2019, version 2306 (assembly 16529.20154), using the add-in «Data Analysis».

### Peat decomposition rate and the type of peat

The peat decomposition rate and the type of peat were studied using the Bio 2 binocular microscope («Altami», Russia), complemented by the digital camera Ucmos 03100KPA and Altami Studio software. The plant remains were indicated according to the established protocol (Mauquoy et al. 2010). The degree of decomposition was determined microscopically in accordance with Drzymulska (2016).

### Water content. Ash content (Z), pH value

Peat water content was found after drying of samples and ash content - after their combustion (Zubov et al. 2022). The pH of the peat deposit was tested *in-situ* by the direct potentiometry on the liquid analyser EXPERT-001 (Econix, Russia) with the combined electrode ESK-10603 attached for pH measurement. The pore water squeezed from the corresponding peat layer was therefore measured.

## Discussion

Table 1 suggests the studied peat core’s botanical composition is highly homogenic, being composed predominantly by *Sphagnum* mosses with an admixture of cotton grass and brown mosses. Containing pine wood residues, the layer of 305-315 is an exception.

The decomposition rate of the bulk of the peat core ranges from 5 to 15% and increases with depth reaching 25–30% if deeper than 250 cm. Generally, the decomposition rate increases with depth, with the highest values observed in the bottom layers.

The ash content of the ombrotrophic peat samples match the one typical for oligotrophic peat and does not exceed 2%. The highest values are normally observed at the bottom (1.4-0.1%) and the surface (1.8-0.1%) layers. In the first case, it is due to the proximity of the mineral underlying rocks and the run-off from nearby areas at the beginning of the bog-formation process. In the second case, it is due to an increase of dry atmospheric deposition (dust and mineral) over the past 50 years. The peat core through the whole depth is composed of ombrotrophic peat and deoxidises with depth. The pH values range from 3.34 to 4.16.

**Figure 3.**
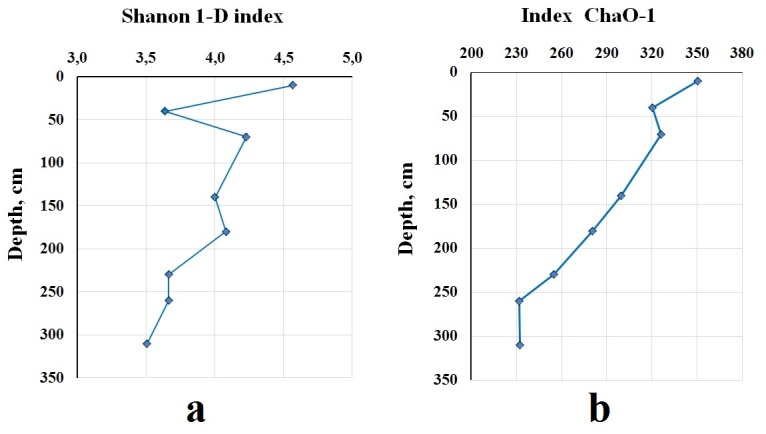
Indices of alpha-diversity of studied peat core: **a** Shanon 1-D; **b** ChaO-1.

**Figure 4.**
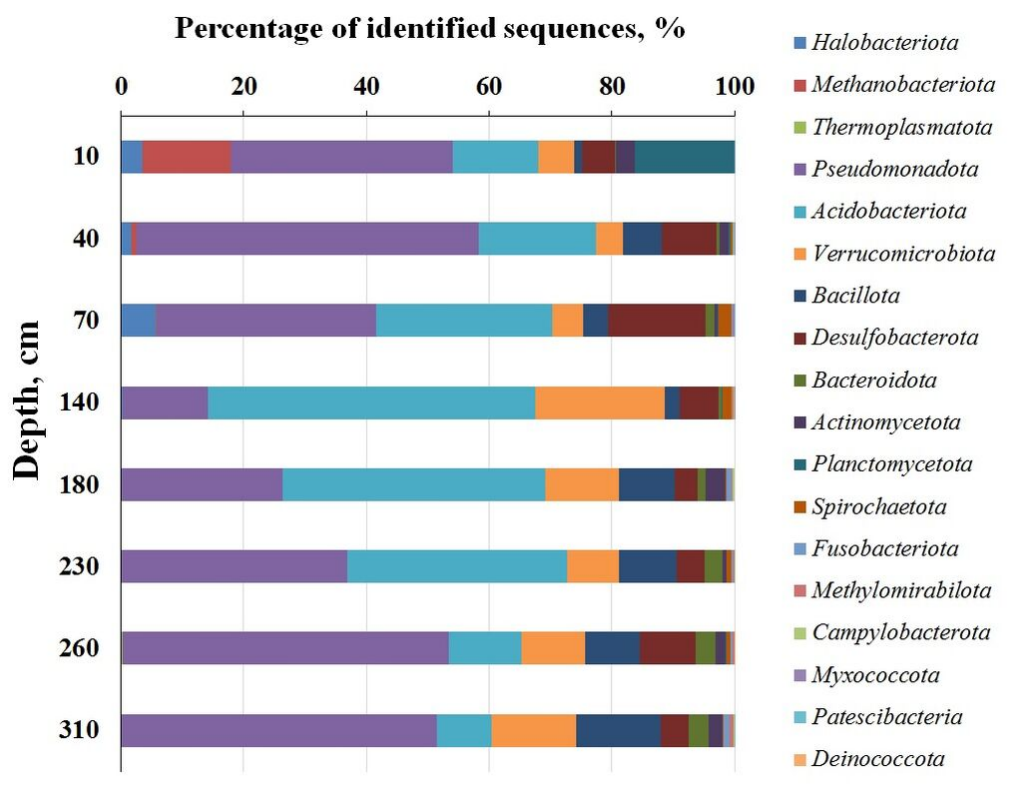
Prokaryotes at the phylum level at Ilas Bog Massif’s peat deposit.

**Figure 5.**
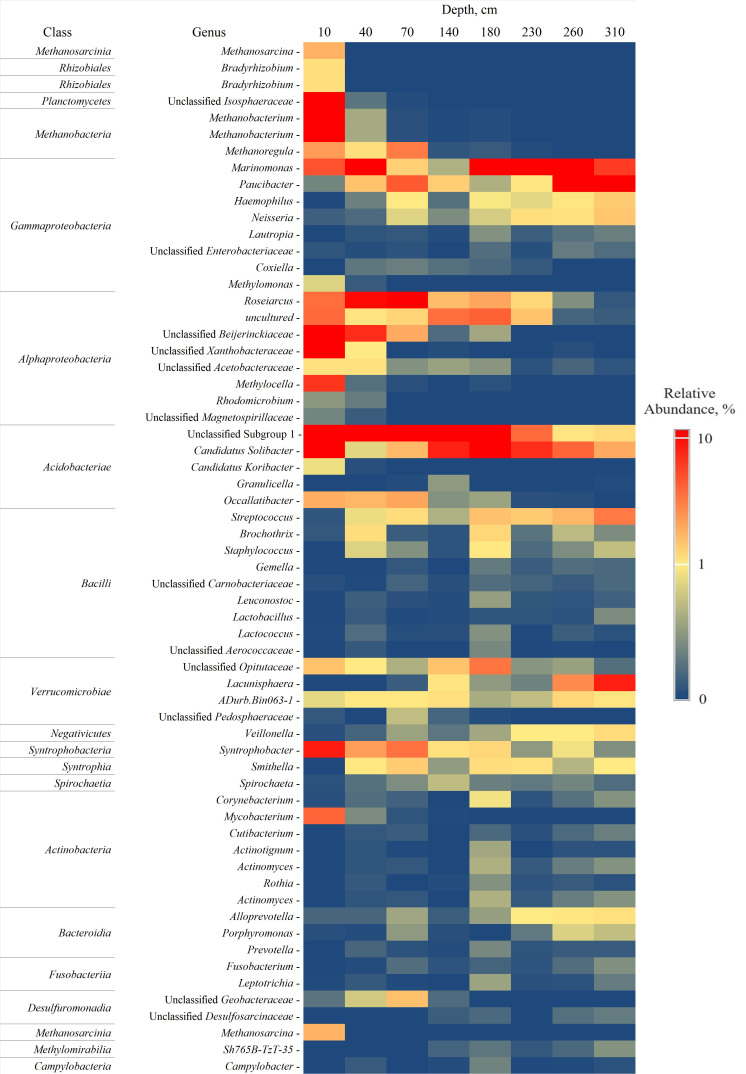
The prokaryotic community at the level of genera (excluding rare genera (< 0.2% of total content)) at Ilas Bog Massif’s peat deposit.

Performance sequencing of 16S rRNA genes produced a total of 19 phylums (105 species) of prokaryotes, of which four are archaea and 15 are bacteria. Eight phyla of bacteria are typical throughout all layers (Fig. 4). Different biodiversity metrics were applied to estimate the composition and diversity of identifiable phyla when analying 16S rRNA gene sequencing data (Kers and Saccenti 2022). In order to reflect the depth dynamics in the study area, we selected the widely used one-dimensional non-parametric alpha-diversity indices Shannon 1-D and ChaO1 (Xia and Sun 2023). Shannon 1-D index ranges from 3.5 to 4.6 in the studied peat core, remaining at a consistently high level (> 2) in all tested layers (Fig. 3). The index is declining by 23.2% with the depth. The moderate dynamics of the Shannon 1-D index between the studied layers is due to the homogeneous composition of the predominant phyla, which is similar in both phylum sets and relative richness. The thermal map shows the relative distribution of families in the peat core (Fig. 3, Fig. 5).

The results of data processing showed that 2,296 taxonomic units have been identified (ChaO1) decreasing with depth by 33.9% with a high level of linear regression (> 0.95) (Fig. 3).

The highest number was observed at the depth of 10 cm: ChaO1 was estimated as 351. It also has the highest Shannon biodiversity (H = 4.6). The lowest value of species richness (H = 3.5) with the number of estimated taxonomic units (ChaO1 = 232) was observed at the 310 cm depth, which makes sense as the upper layer of the deposit is characterised by the maximum variability of key parameters determining the functioning of the microbiota. Seasonal and diurnal variations in temperature and level of bog water, as well as in the organic matter composition of the deposit, create suitable conditions for a wider range of prokaryotes.

The bacterial communities, as mentioned above, show convergence in the predominant phyla throughout the depth of the deposit (Fig. 4). Characterised by high biodiversity, *Pseudomonadota* ranges from 14.0 to 55.8% and predominates in all layers (except for the one between 140 and 180 cm). The amount of *Alphaproteobacteria* decreases (30.7-0.5%) and the amount of *Gammaproteobacteria* increases (4.7-48.8%) with the depth. In the acrotelm, *Alphaproteobacteria* are represented by the order *Rhizobiales*, mainly consisting of the *Beijerinckiaceae*, *Acetobacteraceae* and *Xanthobacteraceae* groups (up to 30% of the total community). The species of these groups exist in different environments and are often found in oligotrophic peat (Kimoto et al. 2010, Serkebaeva et al. 2013). The *Gammaprotmapeobacteria* is almost completely represented by the genus *Burkholderia* of the *Neisseriaceae* family in the middle layers of the peat core (up to 5% bacterial community) and *Comamonadaceae* at the bottom layers (up to 15% community composition). Most of the *Gammaprotmapeobacteria* class consist of bacteria of the genera *Marinomonas* and *Paucibacter*. The genus *Marinomonas* was proposed by Van Landshut and De Lei (1983) and their representatives were found to be common in various marine environments (Van Landschoot and DE LEY 1983). Representatives are known for their adaptation to the environment and metabolic versatility, with a large amount of protein, related to resistance to extreme temperatures and osmotic pressure, as well as carbohydrase and multiple secondary metabolites (Yu et al. 2020). Although *Marinomonas* are primarily aerobic, they occur in the studied deposit at all depths, ranging from 0.6% to 6.7%. The genus *Paucibacter* of the *Comamonadaceae* family was first described by Rapala et al. (Rapala et al. 2005). Although high-quality genomic sequences of 13 strains of *Paucibacter* have been published, no extensive studies of the genomic traits and ecological role of the genus *Paucibacter* have been conducted (Kayani et al. 2022). However, earlier studies indicated the characteristic ability of this genus to decompose microcystines and nodularin (Rapala et al. 2005). Other characteristics include gram-negative wand-shaped cells that are positive to oxidase, weakly positive to catalase and are mobile by means of a single polar flagellum (Pheng et al. 2017).

Another common phylum is the *Acidobacteriota* (4.5-51.5%), presented in all studied layers. Its abundance is reduced due to high biodiversity in the upper aerated and poorly-aerated layers of peat core. This is the predominant phylum in the middle part of the peat core (140-230 cm). Its amount decreases significantly as depth increases and acidity declines. Despite its high abundance, the *Acidobacteriota* has low biodiversity in the peat core, being represented only by three families. The most numerous is one subgroup of the *Acidobacteriaceae* (up to 30%), which plays an important role in the decomposition of cellulose (Pankratov et al. 2011). Earlier, it was reported that the phylum *Acidobacteria* are common in oligotrophic peat with a low nutrient content and have been isolated from numerous objects (Männistö et al. 2012, Serkebaeva et al. 2013, Lin et al. 2014).

The presence of the phylum *Verrucomicrobiota* (4.4-21.1%), represented by the *Verrucomicrobiae* and *Chlamydiae* classes, increases in the middle part of the peat core (sample 140). Most of the sequences formed the group *Opitutaceae* and *Pedosphaeraceae* (up to 17%) (Fig. 5). Previously, representatives of these families were identified in both the surface (Serkebaeva et al. 2013) and in the deep layers (Dedysh et al. 2006) of the oligotrophic *Sphagnum* peat cores. It is known that *Verrucomicrobiae* are enzymatic anaerobes in some oligotrophic peat cores (van Passel et al. 2011) and, therefore, have the ability to decompose the lignocellulose and to fix the nitrogen (Isanapong et al. 2012).

Sequences defined as a phylum *Desulfobacterota* (3.8-16.0%) were assigned to the 4^th^ class: *Desulfobacteria*, *Desulfuromonadia*, *Syntrophia* and *Syntrophobacteria*. Most of the isolated bacteria of the phylum *Desulfobacterota* do not use carbon, making up anaerobic sulphate-reducing populations. The representatives of this phylum are rarely found in oligotrophic peat cores with a low degree of mineralisation, preferring a neutral environment.

The deposits of the *Bacillota* phylum distributed throughout the depth include *Bacilli*, *Clostridia* and *Negativicutes* classes. The last two are presented in some layers in neglible quantities, while the *Bacilli* occur at all depths and increase its content with depth (up to 11% of the total number of sequences). The class *Bacilli* were previously found in oligotrophic peat cores, but unlike our study, was found in surface layers (Dobrovol’skaya et al. 2012, Dobrovol’skaya et al. 2019).

The phylum *Planctomycetota* should be highlighted; it constitutes 16.3% of the total sequence pool in the top 10 cm layer and includes primarily the *Isosphaeraceae* family (hydrolitic potential is the specific feature of the family). Taxonomically characterised representatives are able to dispose of a wide range of polysaccharides of plant and microbial origin (Dedysh and Ivanova 2018). However, some representatives of the *Planctomycetota* are capable of anaerobic oxidation of ammonia to N_2_ (Strous et al. 1999). According to molecular studies, the Isosphaeraceae is one of the numerically predominant groups of Planctomycetota in boreal and subarctic bogs (Moore et al. 2015, Dedysh and Ivanova 2018).

The Archaea are common in the upper layers of the peat core and are represented primarily by *Methanobacteriota* (up to 14.3%) and *Halobacteriota* (up to 5.8%). The archaeal content becomes insignificant and is less than 1% at the depth more than 70 cm The isolated taxa of *Methanomicrobiales*, *Methanobacteriales* and *Methanosareinia* belong to methanogens and show high similarity in ratio and taxonomic composition with the North American bogs in the acrotelm zone (0-70 cm) (Høj et al. 2005, Marusenko et al. 2013).

## Conclusions

Generally, this work shows bacterial and archaeal diversity of the representative ombrotrophic bog in northwest Russia. Using DNA-based metabarcoding, 1195 amplicon sequence variants belonging to 105 genera in peat cores were assigned. The most abundant phyla in samples at different depths are Pseudomonadota and Acidobacteriota. Prokaryote genera were found to differ by depth, for example, *Methanobacterium* and *Methylocella* predominate in the upper layers and are not found at depths over 2 m. It should be noted that a number of ASVs were unidentified, requiring further more detailed studies of the biodiversity of such wetland ecosystems. Based on ChaO1 alpha-diversity metrics, a linear reduction of the number of species with the depth (50–250 cm) of the deposit was established. Thus, the peat deposits of the ombrotrophic boreal bogs exhibit relatively low prokaryote biodiversity, which decreases with depth along with increase of acidophilic microorganisms content. At the same time, the maximum ASV content is characteristic of the acrotelm zone, where the maximum variability of key parameters is ensured in determining the functioning of the microbiota. Furthermore, our datasets will be used to clarify the role of specific microorganisms in the carbon and nitrogen cycles.

## Data resources

The original sequencing output files were placed in the Sequencing Archive Service (SRA) of the BioProject National Biotechnology Information Center (NCBI) database, PRJNA1028248.

## Figures and Tables

**Table 1.** Peat deposit properties.

Sample	Depth, cm	Peat type/admixtures	Decomposition rate, %	Z, %	pH	TBC, cells/g а.d. peat
10	5-15	*Sphagnum* peat/cotton grass	7.9 ± 3.7	1.8 ± 0.1	3.34 ± 0.05	3.41 ± 0.70*109
40	35-45	*Sphagnum* peat/cotton grass	7.9 ± 2.9	1.1 ± 0.1	3.53 ± 0.05	23.70 ± 2.05*109
70	65-75	*Sphagnum* peat/cotton grass	3.9 ± 1.1	0.6 ± 0.1	3.57 ± 0.05	2.94 ± 0.45*109
140	135-145	*Sphagnum* peat/no	4.9 ± 1.5	0.8 ± 0.1	3.75 ± 0.05	2.14 ± 0.01*109
180	175-185	*Sphagnum* peat/brown mosses	8.0 ± 2.8	1.2 ± 0.1	3.84 ± 0.05	1.19 ± 0.01*109
230	225-235	*Sphagnum* peat/cotton grass, brown mosses	8.3 ± 3.8	1.1 ± 0.1	3.94 ± 0.05	1.08 ± 0.03*109
260	255-265	*Sphagnum*-cotton grass/no	22.7 ± 5.6	0.9 ± 0.1	4.04 ± 0.05	0.93 ± 0.03*109
310	305-315	*Hypnum*-*Sphagnum*/pine wood	24.0 ± 3.6	2.4 ± 0.1	4.16 ± 0.05	1.00 ± 0.02*109
